# Association between breast cancer susceptibility loci and mammographic density: the Multiethnic Cohort

**DOI:** 10.1186/bcr2229

**Published:** 2009-02-21

**Authors:** Christy G Woolcott, Gertraud Maskarinec, Christopher A Haiman, Martijn Verheus, Ian S Pagano, Loïc Le Marchand, Brian E Henderson, Laurence N Kolonel

**Affiliations:** 1 Cancer Research Center of Hawaii, University of Hawaii, 1236 Lauhala Street, Honolulu, HI 96813, USA; 2 Department of Preventive Medicine, Norris Comprehensive Cancer Center, Keck School of Medicine, University of Southern California, 1441 Eastlake Avenue, NTT-4436, Los Angeles, CA 90089, USA

## Abstract

**Introduction:**

Mammographic density is a strong risk factor for breast cancer. Our objective was to examine its association with polymorphisms identifying breast cancer susceptibility loci that were ascertained in recent genome-wide association studies.

**Methods:**

Subjects were 825 women who participated in previous case–control studies of mammographic density and genetic factors nested within the Multiethnic Cohort study and were from three ethnic groups (White, Japanese American, Native Hawaiian). Eight polymorphisms (rs2981582 in *FGFR2*, rs3803662 and rs12443621in *TOX3*, rs3817198 in *LSP1*, rs981782 and rs10941679 near *HCN1*/*MRPS30*, rs889312 in *MAP3K1*, and rs13387042 at 2q) were examined. Mammographic density was quantified with a computer-assisted method as the percent dense area: the area of radiologically dense fibroglandular tissue relative to the total breast area that also includes radiologically lucent fatty tissue.

**Results:**

The polymorphism rs12443621 in *TOX3 *was associated with percent dense area; women with at least one G allele (previously associated with increased breast cancer risk) had 3% to 4% higher densities than women with two A alleles. The polymorphism rs10941679 near *HCN1*/*MRPS30 *was also associated with percent dense area; women who were homozygous for the G allele (previously associated with increased breast cancer risk) had 4% to 5% lower densities than women with at least one A allele. The other polymorphisms were not associated with percent dense area.

**Conclusions:**

The available data suggest that the effects of most of these polymorphisms on breast cancer are not mediated by mammographic density. Some effects may have been too small to be detected. The association with rs12443621 may provide clues as to how variation in *TOX3 *influences breast cancer risk.

## Introduction

Mammographic density is related to breast cancer; women within the highest categories of mammographic density are at four to six times higher risk than women within the lowest categories [1]. Mammographic density is conceptualized most often as the percentage of the breast area on a mammogram onto which radiologically dense fibroglandular tissue is projected. The components of this percentage, dense area and breast area, can also be considered, but only dense area and percent dense area are consistently associated with breast cancer risk [1,2].

Mammographic density is influenced by genetics [2-4], with up to 65% of its variation estimated to be due to heritable factors [3,5]. Because breast cancer also has a genetic component [6] and is related to mammographic density, they may share some genetic determinants. Polymorphisms in genes affecting sex hormones, insulin-like growth factors and DNA repair – factors putatively or known to be related to breast cancer risk – have been examined in relation to mammographic density, but few clear and replicated relations have been observed [2,7].

Genome-wide association studies (GWAS) have identified breast cancer susceptibility loci in or near genes such as *FGFR2*, *TOX3 *(formerly known as *TNRC9*), *LSP1*, *HCN1/MRPS30*, *MAP3K1 *and at 2q that had not previously been considered [8-10]. Two recent studies examined some of these loci but found no overall association with mammographic density [11,12]. Both studies included predominantly White women, and one study included only premenopausal breast cancer cases [12]. Our objective was to examine associations between polymorphisms identifying breast cancer susceptibility loci and mammographic density in a sample of premenopausal and postmenopausal women with and without breast cancer from the Multiethnic Cohort (MEC) who were White, Japanese American, and Native Hawaiian. The polymorphisms under study have been previously genotyped in the MEC, have all been identified in GWAS, and either were the most strongly associated with breast cancer (rs2981582 in *FGFR2 *[8], rs3803662 and rs12443621in *TOX3 *[8,9], rs889312 in *MAP3K1 *[8], rs13387042 at 2q [9]), have shown some indication of association with mammographic density in previous studies (rs3817198 in *LSP1 *[11,12]), or are close to a region found to be linked to mammographic density (rs981782 and rs10941679 near *HCN1*/*MRPS30 *[4]).

## Materials and methods

### Study population

Subjects were the subset of women who were included in a case–control study of mammographic density [13,14] and who were also included in studies of genetic susceptibility [8,9,15,16] nested within the MEC [17]. The MEC study is a prospective investigation of lifestyle factors with respect to cancer outcomes [17]. The cohort was assembled between 1993 and 1996, and included people between the ages of 45 and 75 years in Hawaii and Los Angeles who returned a questionnaire including information about ethnicity, weight, menstrual factors, and hormone replacement therapy (HRT) use. Menopausal status at baseline was inferred from reported cessation of menstrual periods or initiation of HRT use [18]. Participants chose all of their applicable racial/ethnic groups from a list of the most common, writing in any others; those reporting mixed ethnicity were assigned to a single group based on the priority ranking: African American, Native Hawaiian, Latina, Japanese American, and White. Women were excluded if they had been diagnosed previously with breast cancer, endometrial cancer, or ovarian cancer [17].

The mammographic density study included MEC participants from Hawaii, and included 607 cases who were diagnosed with invasive breast cancer by the end of December 2000 and 667 controls who were frequency matched to cases in ethnic and 5-year age groups [13]. Participants filled out another questionnaire requesting updated information so that menopausal status, HRT, and weight at the time of each mammogram could be inferred [14].

To enable case–control studies of genetic susceptibility, a blood sample was requested during 1995 to 2000 from breast cancer cases occurring in the MEC and from a random sample of controls, and subsequently from a substantial portion of the MEC participants [15]. These participants have been included in the replication steps of two GWAS [8,9,16]. In these GWAS, approximately 300,000 single nucleotide polymorphisms were genotyped in Caucasian populations, either selected to have a strong family history of breast cancer or not so selected, to identify those that were most highly associated with breast cancer risk under a co-dominant model. The most highly ranked polymorphisms were then tested in several replication sample sets, one of which included cases and controls from the MEC. These studies nested in the MEC included cases diagnosed by the time of the inception of each study and a selection of controls. The MEC subjects sampled for the replication steps of the two GWAS largely overlapped.

The current analysis involved 361 cases and 464 controls who were part of the mammographic density study, had at least one polymorphism of interest genotyped for the replication steps of the GWAS, and were in the major ethnic groups represented in Hawaii (White, Japanese American, Native Hawaiian). The MEC and its genetic substudies were approved by the Institutional Review Boards at the University of Southern California and the University of Hawaii. The mammographic density study was approved at the University of Hawaii. Participants provided informed consent for both case–control studies.

### Mammographic density

Mammograms were available for a mean of 2.8 different dates per woman. Mammograms were performed before diagnosis for all but five cases. For these cases, a mammogram was available only at the time of diagnosis so the film of the contralateral breast was used. Otherwise, if mammograms from both breasts were available on a single date, their measures were averaged.

Films of the craniocaudal view were digitized using a Lumisys 85 scanner (Eastman Kodak, Rochester, NY, USA) that creates images with pixel size equal to 260 μm and has an optical density from 0 to 4.1. Mammographic density was determined using the computer-assisted software, Cumulus, developed at the University of Toronto, Canada [19]. This software allowed the reader (GM) to select two thresholds based on pixel brightness: one to delineate between breast and background, and another to delineate between nondense and dense areas in the breast. Percent dense area was estimated from the dense area divided by the breast area; measurements were very reliable (intraclass correlation = 0.974; 95% confidence interval = 0.968 to 0.978) [13].

### Genotyping

Blood was obtained from subjects at their homes, processed within 8 hours, and stored at -80°C. DNA was purified from white blood cell fractions using the QIAamp 96 DNA Blood Kit (Qiagen, Valencia, CA, USA). The details of the 5'-nuclease assay (Taqman) [8,9] used for most of the assays and the Nanongen Centaurus assay used for rs10941679 [16] have been previously described. For the eight polymorphisms in this analysis, the average concordance for the blinded duplicates was 98.8% (1,044/1,056), with a range of 98.1% to 100%.

### Statistical analysis

Chi-square test statistics for the hypothesis of Hardy–Weinberg equilibrium and the corresponding levels of significance were calculated by ethnic group. Significant deviation from what was expected (*P *< 0.01) only occurred for the rs981782 genotypes among Native Hawaiian subjects (*P *= 0.0008), possibly due to instability arising from small numbers (n = 39).

To investigate the associations between the genotypes and percent dense area, mixed models were applied to account for subjects with multiple mammographic readings over time. This method is nearly equivalent to modeling each woman's average percent dense area over time except that it uses maximum likelihood rather than ordinary least squares estimation. The adjusted mean percent dense area was estimated by genotype. Tests for the difference in mean percent dense area and associated *P *values with each allele previously associated with breast cancer risk (co-dominant, *P*_c_), with any risk allele (dominant, *P*_d_), or with both risk alleles (recessive, *P*_r_) were performed. Adjustment was made for age and the square of age at the time of each mammogram because percent dense area decreases at a rate that slows over time [14], for ethnicity because it is associated with genotype and percent dense area, and for body mass index at the time of each mammogram because it strengthens the association between percent dense area and breast cancer risk [20]. Variables for which adjustment was not made in the models included reproductive characteristics because they could be on the causal pathway, family history of breast cancer because it could be a proxy for genotype, case status because it is theoretically downstream of mammographic density, and HRT because it was not associated with any of the genotypes. Variables for which an interaction with the genotypes was investigated, based on previous literature [7,11,21,22], included case status, ethnicity, and menopausal status with HRT. The absolute amount of dense area was investigated in similar models. All *P *values are two-sided with the level of significance set at *P *< 0.05. SAS 9.1 (SAS, Cary, NC, USA) was used for the analysis.

## Results

For each polymorphism of interest, 634 to 806 subjects were genotyped; 825 subjects had at least one of the polymorphisms genotyped. Characteristics of these 825 subjects are presented in Table 1. The women had mean ± standard deviation age of 59.5 ± 8.8 years and body mass index of 25.3 ± 5.5 kg/m^2^. Women were of White (32%), Japanese American (44%) or Native Hawaiian (24%) ethnicity. At the time of the first mammogram used in this study, 201 (24%) of the women were premenopausal; because subjects had multiple mammographic readings over time, a lower proportion (18%) of mammograms used was taken when the subjects were premenopausal. The mean percent dense area was 32.7 ± 22.2.

**Table 1:** Selected characteristics of the 825 participants

Characteristic	Value
Ethnicity	
White	262 (31.8%)
Japanese American	361 (43.8%)
Native Hawaiian	202 (24.5%)
Menopausal status, hormone replacement therapy at first mammogram	
Premenopausal	201 (24.4%)
Postmenopausal, no hormone replacement therapy	204 (24.7%)
Estrogen only hormone replacement therapy	228 (27.6%)
Estrogen and progestin hormone replacement therapy	192 (23.3%)
Case status	
Case	361 (43.8%)
Control	464 (56.2%)
Family history (mother or sister)	
Yes	114 (13.8%)
No	711 (86.2%)
Mean age over all mammograms (years)	59.5 ± 8.8
Mean body mass index over all mammograms (kg/m^2^)	25.3 ± 5.5
Mean percent dense area (%)	32.7 ± 22.2
Mean dense area (cm^2^)	32.5 ± 25.4
Mean breast area (cm^2^)	117.1 ± 60.0

Data presented as *n *(%) or mean ± standard deviation. The set of 825 subjects is the union of the individual sets of 634 to 806 subjects who were genotyped for each polymorphism of interest; that is, 825 subjects had at least one polymorphism of interest genotyped.

**Figure 1:**
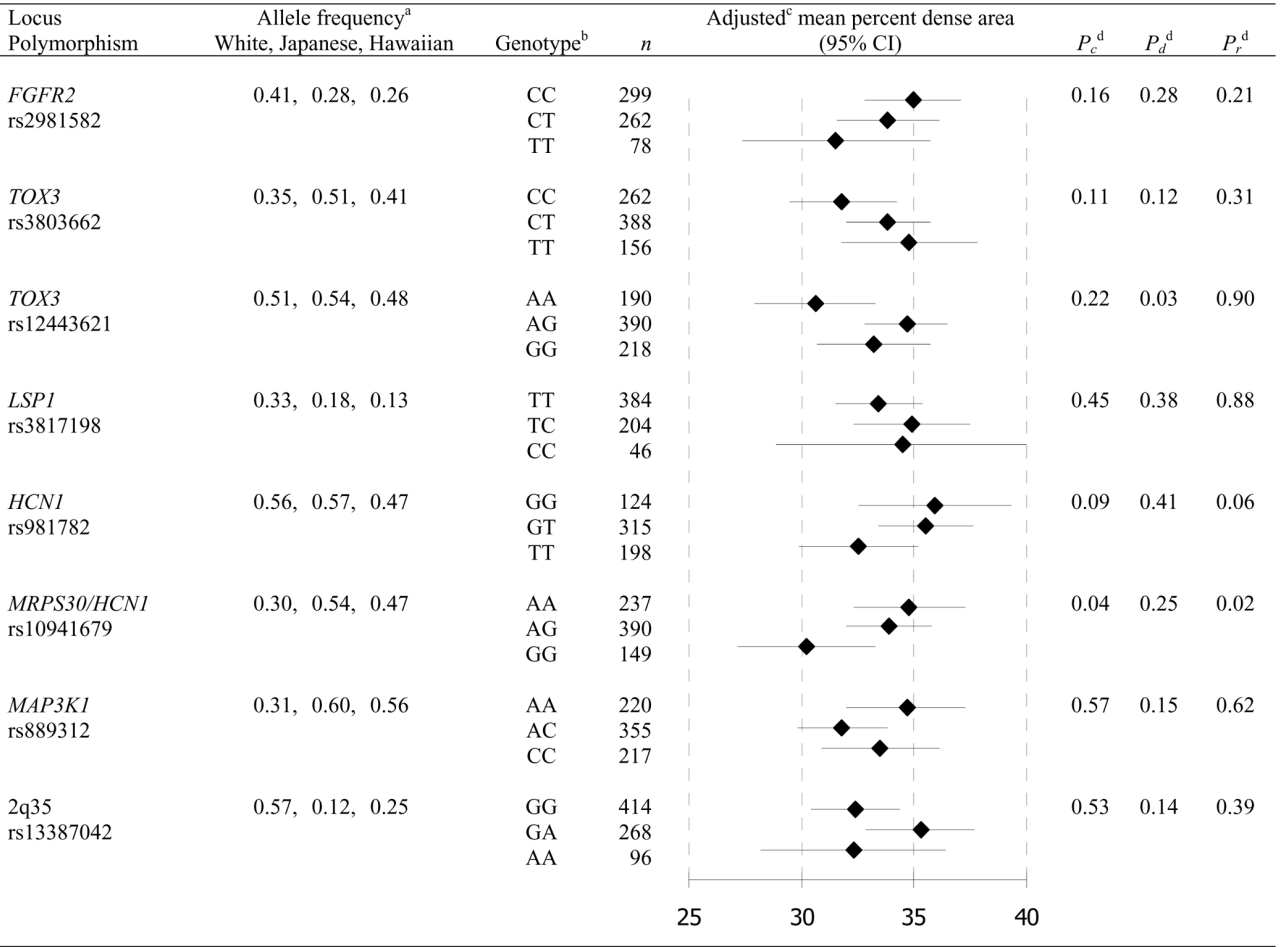
Adjusted mean percent dense area by genotype. ^a^Frequency of the breast cancer risk allele. ^b^In order of increasing breast cancer risk observed in previous studies [8,9,16]. ^c^Adjusted for age, age squared and body mass index at mammogram and ethnicity. Numeric data are shown in Additional data file 1. ^d^*P*_c_, co-dominant *P *value for the per-allele increase in percent dense area; *P*_d_, dominant *P *value for an increase in percent dense area with any risk allele; *P*_r_, recessive *P *value for an increase in percent dense area with both risk alleles. CI, confidence interval.

Having any G allele of rs12443621 in *TOX3 *was associated with a significantly higher percent dense area than being homozygous for the A allele (*P*_d _= 0.03) (Figure 1 and Additional data file 1). This association was modified by menopausal status with HRT (*P*_interaction _= 0.049) and seemed to be more pronounced in premenopause. This association was nonsignificantly stronger in cases than in controls (*P*_interaction _= 0.054); with dense area, however, the association was significantly stronger in cases and no association was evident in controls (*P*_interaction _= 0.01). Furthermore, among Whites homozygotes of the G allele had a higher absolute dense area, among Native Hawaiians homozygotes of the G allele had a lower absolute dense area, and no association was observed among Japanese Americans (*P*_interaction _= 0.002).

Being homozygous for the G allele of rs10941679 near *HCN1/MRPS30 *was associated with a significantly lower percent dense area than being having any A allele (*P*_r _= 0.02) (Figure 1 and Additional data file 1). This association was nonsignificantly stronger in cases than in controls (*P*_interaction _= 0.12); with dense area, the association was significantly stronger in cases (*P*_interaction _= 0.02).

Percent dense area and dense area were not significantly associated with any other polymorphism overall and no other interactions were detected with either case status or ethnicity (*P*_interaction _> 0.05). Menopausal status with HRT significantly modified some associations, suggesting that, in users of combined HRT, being homozygous for the T allele of rs2981582 in *FGFR2*, being homozygous for the T allele of rs981782 in *HCN1*, or being homozygous for the A allele of rs13387042 at 2q35 was associated with decreased dense area (*P*_interaction _= 0.02 for all), and each C allele of rs3817198 in *LSP1 *was associated with increased percent dense area (*P*_interaction _= 0.003).

## Discussion

The rationale for this investigation was that breast cancer susceptibility loci may also be related to another strong risk factor, mammographic density. Two polymorphisms examined were associated with percent dense area. Women with any G allele of rs12443621 in *TOX3 *had 3% to 4% higher percent dense area than women homozygous for the A allele; the association was stronger in premenopausal women and breast cancer cases. Previously, each G allele has been found to be associated with increased breast cancer risk [8], whereas we found that a dominant model fit best in explaining variability in mammographic density. In an investigation nested within the Nurses' Health Study, premenopausal women homozygous for this allele had higher percent dense area and dense area, but postmenopausal women did not [11]. The polymorphism rs10941679 near *HCN1 *and *MRPS30 *was also associated with percent dense area; women who were homozygous for the G allele had 4% to 5% lower densities than women with at least one A allele. The G allele, however, has been found to be associated with increased breast cancer risk in a previous study under a co-dominant model [16]. This polymorphism has not been examined in previous studies of mammographic density. Other polymorphisms identifying breast cancer susceptibility loci were not associated with percent dense area or dense area in the overall samples in the current study or in two other published studies [11,12]. All studies, however, found that the C allele of rs3817198 in *LSP1 *was associated with higher percent dense area in subgroups of their samples: in premenopausal women [11], in steroid receptor-positive cases [12], or in current users of combined HRT in this study.

The polymorphisms examined in the present study were identified in GWAS of breast cancer [8,9]. The strongest associations have been found with polymorphisms in *FGFR2*; each copy of the T allele of rs2981582 was found to be associated with a 26% increased breast cancer risk [8]. Because fibroblast growth factors and their receptors are thought to provide a mechanism for epithelial–mesenchymal interactions [23] and because mammographic density is largely a reflection of the amount of dense stromal tissue that may provide a permissive environment for neoplastic transformation of the epithelial cells, we had hypothesized that variation in *FGFR2 *would be related to mammographic density. In this study, however, percent dense area was nonsignificantly decreased with the number of risk alleles.

Polymorphisms in or near *TOX3*, *LSP1*, *HCN1*, *MAP3K1 *and at 2q35 are less strongly associated with breast cancer risk than polymorphisms in *FGFR2*; the increased risks range from 4% to 20% with each risk allele [8,9,16,24,25]. *TOX3 *contains a high mobility group box motif that suggests it is a transcription factor [8], but the specific mechanism by which variation in *TOX3 *affects breast cancer risk is unknown. Our results and those of others [11] suggest that mammographic density could be an intermediate factor, but confirmation from other studies is needed. We observed a stronger association in cases; it could be that cases have other environmental or genetic factors interacting with *TOX3 *to increase both mammographic density and breast cancer risk. *HCN1 *is adjacent to other genes that may be related to breast cancer risk including *FGF10 *and *MRPS30 *[16], and is just outside a region linked to mammographic density [4]. *LSP1*, lymphocyte-specific protein 1, may be involved in wound healing [26], which involves some processes in common with mammary gland development and involution that could contribute to fibrosis and, thus, mammographic density [27,28]. Some aspects about the biology of these genes suggest that polymorphisms in or near them could affect mammographic density. On the other hand, some reports suggest that variation in *FGFR2*, *TOX3*, *HCN1*, and at 2q35 may be more strongly associated with estrogen receptor-positive cancers [9,16,25], but mammographic density has not been found to be differentially related to breast cancers by steroid receptor status [29].

A limitation of the current study was that the sample size was insufficient to detect interactions with case status, ethnicity, and menopausal status with HRT or to detect small effects. With the sample size in this study, a linear increase per allele of 2% to 3% percent dense area could be detected with 80% power. If an allele increases breast cancer risk between 4% and 26% [8,9,16] and if the increased risk was entirely mediated by mammographic density, percent dense area would be expected to be 2% to 13% higher with each allele (an increase of 1% dense area increases breast cancer risk by approximately 2% [1]). Differences may not have been detectable if less than the entire effect was mediated through mammographic density. Some of the polymorphisms studied seem to be more strongly related to breast cancer susceptibility in Whites than Asians [8] and the true effect may have been smaller in our overall study population, of which nearly one-half was Japanese American. Furthermore, the causative variants for breast cancer represented by the polymorphisms identified in GWAS have not been identified [16] but could be related more strongly to both breast cancer risk and mammographic density. Note that no results in the present study would have been statistically significant if correction had been made for multiple testing. Whether they were chance findings will need to be determined by replication in other studies.

Selection bias was unlikely to mask associations. Although our sample was a subset of the MEC with a slightly different age and ethnic distribution, we controlled for these factors in the analysis; other unknown or unmeasured factors that influenced selection into this analysis are probably less likely than age or ethnicity to be related to either genotypes or mammographic density. The association between the polymorphisms and mammographic density is therefore unlikely to have been modified by these factors.

## Conclusions

The rs12443621 polymorphism in *TOX3 *was associated with percent dense area under a dominant model. Some biologic plausibility exists for associations with the other polymorphisms examined but possibly the associations were too small to be detected. The available data, however, suggest that the effects of most of these polymorphisms on breast cancer are not mediated by mammographic density. The association of mammographic density with rs12443621, if confirmed, may provide clues as to how variation in *TOX3 *influences breast cancer risk.

## Abbreviations

GWAS: genome-wide association study; HRT: hormone replacement therapy; MEC: Multiethnic Cohort.

## Competing interests

The authors declare that they have no competing interests.

## Authors' contributions

CGW established the concept of this project, carried out the statistical analysis, interpreted the results, and wrote the manuscript. GM established the mammographic density study and provided input into the interpretation of the data. CAH provided input into the study concept and the interpretation of results, and was involved in the parent genetic susceptibility studies. MV provided input into the study concept, statistical analysis, and interpretation of results. ISP performed initial data manipulation in the mammographic density study and provided expertise in the statistical analysis. LLM provided input into the study concept, statistical analysis and interpretation of results, and was involved in the parent genetic susceptibility studies. BEH and LNK are the Principal Investigators of the MEC. Furthermore, all authors provided feedback on the initial draft of the manuscript and approved the final manuscript.

## Supplementary Material

Additional file 1 An Adobe file containing the table "Adjusted mean percent dense area and dense area by genotype." Click here for file
