# Supplementary material for: Association between breast cancer susceptibility loci and mammographic density: the Multiethnic Cohort
Source: Breast Cancer Res. 2009 Feb 21;11(1):R10. doi: 10.1186/bcr2229 (PMC2687715; doi:10.1186/bcr2229)
Supplement: Additional file 1 — An Adobe file containing the table "Adjusted mean percent dense area and dense area by genotype." [file bcr2229-S1.pdf]

Supplementary file: Adjusted mean percent dense area and dense area by genotype

Locus			Percent dense area					Dense area (cm^2)				
Polymorphism	Genotype[a]	*n*	Mean[b]	(95% CI)	P_c	P_d	P_r	Mean[b]	(95% CI)	P_c[c]	P_d[c]	P_r[c]
FGFR2	CC	299	35.0	(32.8-37.1)	0.16	0.28	0.21	32.2	(29.7-34.8)	0.27	0.47	0.23
rs2981582	CT	262	33.8	(31.6-36.1)				31.6	(29.0-34.3)			
	TT	78	31.5	(27.4-35.7)				28.8	(23.9-33.6)			
near *TOX3*	CC	262	31.8	(29.5-34.2)	0.11	0.12	0.31	31.9	(28.9-35.0)	0.43	0.63	0.40
rs3803662	CT	388	33.8	(32.0-35.7)				32.4	(29.9-34.9)			
	TT	156	34.8	(31.8-37.8)				34.1	(30.2-38.0)			
TOX3	AA	190	30.6	(27.9-33.3)	0.22	0.03	0.90	29.3	(25.8-32.8)	0.12	0.06	0.49
rs12443621	AG	390	34.7	(32.8-36.5)				33.2	(30.7-35.7)			
	GG	218	33.2	(30.7-35.7)				33.3	(30.0-36.6)			
LSP1	TT	384	33.4	(31.5-35.4)	0.45	0.38	0.88	30.4	(28.0-32.9)	0.15	0.07	0.99
rs3817198	TC	204	34.9	(32.3-37.5)				34.6	(31.2-38.0)			
	CC	46	34.5	(28.9-40.0)				32.0	(24.8-39.2)			
HCN1	GG	124	35.9	(32.5-39.3)	0.09	0.41	0.06	33.2	(28.8-37.7)	0.40	0.69	0.34
rs981782	GT	315	35.5	(33.4-37.6)				33.0	(30.2-35.8)			
	TT	198	32.5	(29.9-35.2)				31.1	(27.6-34.5)			
MRPS30/HCN1	AA	237	34.8	(32.3-37.3)	0.04	0.25	0.02	33.4	(30.1-36.7)	0.32	0.69	0.21
rs10941679	AG	390	33.9	(32.0-35.8)				33.3	(30.8-35.8)			
	GG	149	30.2	(27.2-33.3)				30.4	(26.3-34.5)			
MAP3K1	AA	220	34.7	(32.0-37.3)	0.57	0.15	0.62	33.1	(29.9-36.2)	0.87	0.42	0.60
rs889312	AC	355	31.8	(29.8-33.8)				31.0	(28.6-33.3)			
	CC	217	33.5	(30.9-36.1)				32.6	(29.5-35.7)			
2q35	GG	414	32.4	(30.4-34.4)	0.53	0.14	0.39	32.4	(29.8-35.1)	0.80	0.58	0.21
rs13387042	GA	268	35.3	(32.9-37.7)				34.4	(31.3-37.5)			
	AA	96	32.3	(28.2-36.4)				30.0	(24.5-35.5)			

[a] In order of increasing breast cancer risk observed in previous studies [8,9,16].
[b] Adjusted for age, age^2 and body mass index at mammogram and ethnicity (Caucasian, Japanese, Native Hawaiian).
[c] P_c: co-dominant *P*-value for the per-allele increase in mammographic density; P_d: dominant *P*-value for an increase in mammographic density with any risk allele; P_r: recessive *P*-value for an increase in mammographic density with both risk alleles.

CI, confidence interval.
